# Applying the institutional review board data repository approach to manage ethical considerations in evaluating and studying medical education

**DOI:** 10.3402/meo.v21.32021

**Published:** 2016-07-20

**Authors:** Erin K. Thayer, Daniel Rathkey, Marissa Fuqua Miller, Ryan Palmer, George C. Mejicano, Martin Pusic, Adina Kalet, Colleen Gillespie, Patricia A. Carney

**Affiliations:** 1 Department of Family Medicine, Oregon Health & Science University, Portland, OR, USA; 2 Department of Medicine, Oregon Health & Science University, Portland, OR, USA; 3 Division of Learning Analytics, Institute for Innovations in Medical Education, NYU School of Medicine, New York, NY, USA; 4 Department of Emergency Medicine, NYU School of Medicine, New York, NY, USA; 5 Medical Education Outcomes Unit, Program for Medical Education Innovation and Research, NYU School of Medicine, New York, NY, USA; 6 Division of Evaluation and Outcomes, Institute for Innovations in Medical Education, NYU School of Medicine, New York, NY, USA; 7 Department of Medicine, Institute for Innovations in Medical Education, NYU School of Medicine, New York, NY, USA

**Keywords:** educational research, ethical review, program evaluation, longitudinal assessment

## Abstract

**Issue:**

Medical educators and educational researchers continue to improve their processes for managing medical student and program evaluation data using sound ethical principles. This is becoming even more important as curricular innovations are occurring across undergraduate and graduate medical education. Dissemination of findings from this work is critical, and peer-reviewed journals often require an institutional review board (IRB) determination.

**Approach:**

IRB data repositories, originally designed for the longitudinal study of biological specimens, can be applied to medical education research. The benefits of such an approach include obtaining expedited review for multiple related studies within a single IRB application and allowing for more flexibility when conducting complex longitudinal studies involving large datasets from multiple data sources and/or institutions. In this paper, we inform educators and educational researchers on our analysis of the use of the IRB data repository approach to manage ethical considerations as part of best practices for amassing, pooling, and sharing data for educational research, evaluation, and improvement purposes.

**Implications:**

Fostering multi-institutional studies while following sound ethical principles in the study of medical education is needed, and the IRB data repository approach has many benefits, especially for longitudinal assessment of complex multi-site data.

Ethical considerations in evaluation of medical education can be complex. For example, in 2003 several US medical schools and the Association of American Medical Colleges (AAMC) came under investigation regarding the administration and use of the AAMC Graduation Questionnaire (GQ). Some medical schools made completion of the GQ a graduation requirement, and either refused to refund students’ financial deposits until they completed the questionnaire or used other approaches to improve response rates (1). Obtaining a sample representative of US medical school graduates would allow researchers to publish articles examining trends in medical education (2). However, a group of medical students, along with their attorneys, accused these medical schools of coercion and the AAMC of unethical publication of students’ data without institutional review board (IRB) review or approval. Subsequently, an IRB review was undertaken by the AAMC and respective medical schools to resolve the issue.

Since the original Declaration of Helsinki in 1964, ethical approval has been required to ensure ethical standards are upheld in human subjects research (3–5). Further, students at any level are considered vulnerable subjects (5), as they could perceive an increased likelihood of receiving more favorable grades, recommendations, or promotion in class rankings when participating in special assessment activities.

According to the US Code of Federal Regulations (45 CFR 46. 101(b)), educational program assessments and quality improvements, including educational tests, survey procedures, or behavioral observations, can be done at any institution without IRB oversight because these are part of every educational program designed to benefit both educators and learners (5, 6). While IRB oversight may not be needed, a determination of whether the evaluation activity is considered human subjects research is needed. This is important because most journals now require IRB determination prior to review and potential publication of any educational evaluation, regardless of whether it is considered evaluation or research. However, it is not always easy to distinguish between standard evaluative activities and educational research, which likely contributed to the confusion that occurred around the AAMC GQ.

In addition, the Family Educational Rights and Privacy Act (FERPA), a federal law that protects the privacy of student records, applies to all schools that receive US Department of Education funds (7). Thus, medical educators and researchers must ensure students’ rights are respected by complying with both IRB and FERPA regulations (6).

In this paper, we provide an overview of IRB issues as they relate to evaluation and research in medical education, and we propose an IRB repository approach for ethics approval, which is being used by both of the medical schools profiled in this paper. Such an approach is becoming more relevant as innovations in curricular redesigns are occurring across undergraduate and graduate medical education and many of these require longitudinal assessments of learners to determine program effectiveness (8).

## IRBs and educational evaluation and research

Although the same federal laws govern IRBs in the United States, the organization and structure of IRBs are unique to each institution. Many IRBs are more experienced with biomedical research, such as those done in basic and clinical sciences, than they are with social science or educational research. Studies conducted in 2007 (9) and 2013 (10) examined variability in IRB reviews by submitting the same medical education research proposal to multiple institutions across the country. Significant inconsistencies were found in the type of review required, the time it took to review protocols, and modifications requested by the various IRBs studied, even though the protocols submitted were identical (9–11). Some institutions have addressed this issue by creating separate IRB committees for specific research areas. For example, the University of Wisconsin has four separate IRBs, all of which focus only on social science research (Health Sciences IRB, Minimal Risk IRB, Social and Behavioral Sciences IRB, and Educational Research IRB) (12).

IRBs can grant exemptions, approval with waivers of consent or approval with consent based on the examples provided (Table 1). Expedited IRB reviews can occur when the research involves no more than minimal risk to the subjects, as typically occurs in educational settings. Research considered to be more than minimal risk to subjects, such as a study of recreational drug or alcohol use among medical students, necessitates full IRB review, which involves assessment of all study plans, including the consent form (6).

**Table 1:** Delineation of ethical categories in educational research according to the Code of Federal Regulations (45 CFR 46 101(b))

Human subjects involvement	Ethical category	Definition	Example
No human subjects	Exempt after expedited review	Data is factual, program-level data, not learner level even if obtained from a person	USMLE Step 1 and 2 exam pass rates
Human subjects	*Exempt or approved after review with waiver of consent or required consent*	*Determination is based on risk to learners and need to inform learners*	
	Very low risk – likely to be exempt after expedited review	Data collected presents minimal risk to the person	Collecting de-identified student data
	Low risk – may be exempt or approved with waiver or consent after expedited review	Data collected is considered normal educational practice. However, an unproven program may require an information sheet to inform students	Implementing new unproven instructional method and assessing its effectiveness – student data not de-identified
	High risk – approved with informed consent after full board review	Data collected presents more than minimal risk, or those of a sensitive matter, where a breach of confidentiality could be deleterious to the learner	Study on recreational drug use among medical students

Additionally, medical education often employs qualitative research methods. Such methods may complicate qualifying the research as exempt because qualitative studies often use focus groups or interviews that are video or audio recorded and can require sophisticated processes to ensure anonymity. Also, since qualitative research can follow a variety of paths, as occurs when probes are added to key informant interviews, it can be difficult to fully characterize every aspect of the evaluation approach making it difficult to qualify as an IRB exempt activity (6, 13).

## Approach: data repositories as a potential solution

Originally intended to store biospecimens data, repositories are an approach to collect, store, and share data for research purposes (14). The Federal IRB research data repository compliance regulations of the US Department of Health and Human Services have existed since 1997, with updates issued in 2004 and 2010 (14). Justification for a data repository includes an intention that the data (and/or biological specimens) will be used repeatedly for research purposes, stored for future research, and/or shared with other investigators or when there is no explicit plan to discard data when the project is finished (15). This approach facilitates data sharing, increases the use of existing data, and eases ongoing compliance requirements for continuing ethical review. Further, educational researchers can share and pool data with other schools if this is described under the repository scope and laid out in the original repository protocol.

We found six data repositories (16–21) through an initial internet search and subsequent comprehensive literature review in OVID Medline and PubMed using the search terms: data repository, data registry, database, medical students, medical graduates, medical school, medical education, education, outcomes, and tracking. These six repositories exist in medical education for evaluative, quality improvement, administrative tracking, and research purposes. However, when exploring these repositories in detail, we found their approaches to managing ethical considerations varied because their purposes differed. We excluded two repositories because they were used primarily for administrative, tracking, and internal evaluation purposes only with no intent to analyze or publish findings. These include the Tufts University Sciences Knowledgebase (16) and the Canadian Post-MD Education Registry (17). While following their respective institutional policies to protect personal information, these two repositories did not apply for research ethics approval (22, 23). A third repository, based in Australia, the Medical Schools Outcomes Database (MSOD), consists of longitudinal assessment of learners and outcomes tracking data, which has been published in several papers (18). In this case, MSOD obtained consent from all participants and received ethics review from the Australian Institute of Health and Welfare (AIHW) Ethics Committee and each of 18 Australian and 2 New Zealand medical schools involved (24). While the AIHW Ethics Committee has a section for linking educational data, active consent of learners is required (25), and we could not determine that a repository option exists as part of their ethics review process (26). Additionally, according to the Privacy Act 1988, waivers of consent are only granted by the AIHW Committee (via Section 95) if the research is considered *health* research and where the research benefits outweigh the privacy breach (25).

## Examples of existing data repositories using a registry IRB approach

### NYU School of Medicine Registry

New York University (NYU) School of Medicine has created a repository that includes medical students, residents, and fellows. The Research on Medical Outcomes (ROMEO) registry houses consenting trainees’ routinely collected educational data and compiles it in a confidential, longitudinal database to facilitate evaluation and medical education research (19). Data are included in the registry if 1) it is routinely collected as part of the trainee's educational experience; 2) it is collected on *all* trainees with access to the same curriculum; and 3) the trainee has actively consented to allow *identified* data in registry. Safeguards, including a registry monitoring committee, exist to ensure confidentiality of data. Investigators who wish to use data from ROMEO submit a data request and, once approved, are supplied de-identified data from the registry.

ROMEO has been in operation since 2008 with average consent rates of 86% for UME and 71% for residency programs. When residents are approached face-to-face in group meetings or conferences, the consent rates increase to over 90%, but this drops when consent is sought by email. In ROMEO's first 7 years, 72 studies have been published by 51 investigators using these data. As of February 2016, the registry contains data from 2066 individuals, 183 of them have data from both UME and GME. ROMEO's informatics infrastructure has evolved over time (e.g., data collection went from paper through a variety of online data collection tools) with the most significant resource being human efforts required to maintain data quality. Three staff members collect, clean, organize, and report data from ROMEO, all under the oversight of a team of educational researchers. This work is entirely funded by non-institutional sources.

### MedEdNet

Based at Oregon Health & Science University (OHSU), MedEdNet is an AHRQ designated educational research network that involves studies in undergraduate and graduate medical education (20). OHSU successfully obtained an IRB exemption for the MedEdNet repository, which houses data from many educational research studies. The IRB repository application described pooling linkable but de-identified data (a unique study identifier replaces participants’ names) from several studies within the network, including three residency-training studies with approximately 40 primary care residency-training programs (family medicine, general internal medicine, and general pediatrics) with thousands of residents. Surveys from these three studies collect data on residents during and after training, faculty, and clinic staff, with many common variables such as quality of residency training, clinic electronic health record utilization status, resident involvement in quality improvement or research projects, and scope of post-graduate practice. Further, this repository hosts a viewing portal for participating programs that allows them to compare their own program data to aggregate data from other programs in the network. An expedited review with an exemption was obtained from the OHSU IRB using the data repository approach, where each new study was added to the repository through a modification to the original application. Active resident consent was required for only one sub-study, which involved release of In-Training Exam and Board Certification Scores from the American Board of Family Medicine. Importantly, all residency programs also had to undergo IRB review at their respective institutions to provide data to MedEdNet.

The MedEdNet data repository allows for research within and across multi-institutional studies with similar variables and has resulted in over 30 publications. For example, one MedEdNet study examined associations between innovations in 14 geographically diverse family medicine residency-training programs and improvements in the match (27). This study revealed that programs implementing individualized training as part of their curricular innovations significantly improved the percent of positions filled in the match relative to those who did not implement this innovation (90.1% vs. 83.5%, *P*=0.04) (27). In another study, we examined sources of and amounts of funding attained to support educational innovations in residency training, and learned that all but 2 of the 14 participating programs successfully attained funding to support their work and that university-based or administered training programs used different sources of funds relative to community-based programs (28).

### REDEI system

In 2014, OHSU created another repository, funded in part by the American Medical Association (AMA) through the Accelerating Change in Medical Education initiative, which received an IRB exemption with a waiver of consent, though the IRB did require approval of letters sent to students describing the Research and Evaluation Data for Educational Improvement (REDEI) system, its purposes and informing them that evaluation data on them could be included, in aggregate only, in published educational evaluation/research articles (21).

The REDEI system is designed to facilitate real-time peer-comparison feedback to students throughout their medical school education as a way to help them navigate their learning experiences. REDEI also serves as a feedback tool for faculty coaches who are responsible for guiding medical students’ progression through an individualized curriculum, while additionally constituting a data warehouse for educational research using rigorous observational study designs, such as those used in educational epidemiology (29). For example, a component of OHSU's new curriculum effort includes an optional pre-matriculation program that incoming medical students can undertake in July, approximately 6 weeks before the start of medical school. OHSU assessed the extent to which this pre-matriculation program influenced participants’ confidence about entering medical school among those who chose to undertake the pre-matriculation program. We determined that 49.5% reported feeling more confident, 44.1% felt it did not affect their confidence, and 6.3% indicated that they felt less confident. A manuscript describing these findings in detail is currently under development.

Both OHSU and NYU are two schools participating in the AMA's Accelerating Change in Medical Education initiative. There is a plan to define a set of common core variables that will eventually be sent to a master database, located at the AMA for educational research purposes. Approval for this data sharing was included in OHSU's IRB repository application. Amassing a comprehensive common dataset among 11 medical schools provides an unprecedented opportunity for educational research studies, especially if linkages to vital outcome variables can be included in analyses. This approach will allow for multiple pilot analyses that might not have been possible if separate applications were required. OHSU and NYU are also two of the schools participating in the AAMC Core EPAs (Entrustable Professional Activities) for Entering Residency pilot initiative, and this project was added to the REDEI repository application through a modification request, rather than initiating a new IRB application.

## Creating a medical education data repository

When preparing an IRB data repository application, it is important to make conceptual distinctions among data collection and management processes, including: 1) identifying primary data collection at your own institution and others; 2) specific plans for data archiving and sharing, which can occur longitudinally; and 3) data retrieval from the repository for analytic purposes, which, depending on the nature of the repository data, may or may not require IRB review by the retrieving entity (Table 1).

**Fig. 1:**
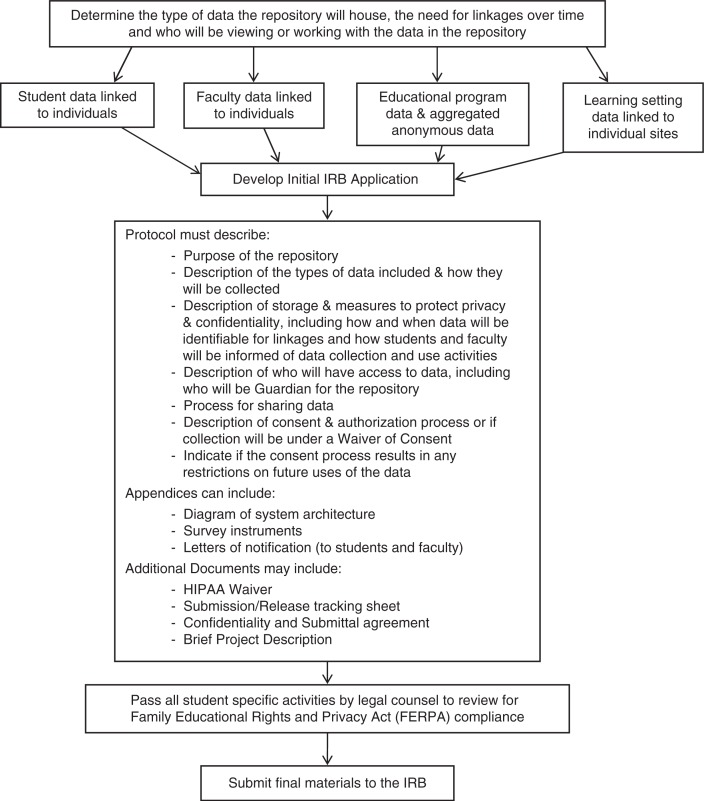
Creating a medical education data repository.

The first step in creating a medical education data repository (Fig. 1) entails determining the type of data the repository will house, the need for linkages over time, and who will be accessing the data in the repository. The second step involves assembling the IRB application, providing a detailed description of the purpose, function, and processes of the system as a whole, including processes for collecting and sharing data, types of data included (including the specific variables being collected, and descriptions of the learning setting and data sources), measures to protect privacy and confidentiality of stored data, access to the data, and guardianship for the repository.

Additionally, the protocol should include a detailed explanation of how the proposed repository and data that it will house meet requirements for a waiver of learner consent via the Code of Federal Regulations for the Protection of Human Subjects (45 CFR 46.102 (h)(i)). Often this requires a letter informing learners how their de-identified data will be used. Application appendices can include any additional materials central to repository functionality, such as a diagram of system architecture or function, a collection of survey instruments, and notification letters to students informing them of the use of their assessment and evaluation data. Additional documents may include a HIPAA waiver of consent, submission/release tracking sheet (to track input and output of data), confidentiality and submittal of data use agreements (form to obtain permission from other institutions for use of their data), and brief project description. Prior to submission of the IRB application, the respective institution's legal council should review the elements involving students to ensure evaluation and data use plans are consistent with FERPA.

## Discussion

Conducting large-scale educational research is challenging when considering tightly scheduled medical school curricula, medical students and residents who are continuously engaged in patient-care responsibilities, and diverse educational settings such as hospitals, clinics, and medical centers that face varying regulatory and financial pressures. In addition, submitting multiple separate IRB applications to cover evaluation activities or educational research on several topics can be a challenge for conducting critical exploratory research needed to guide educational policy and practice.

The data repository approach to ethical review for medical education research has many benefits, including efficiencies with data management and analysis and seamless collaboration within and across institutions. Having a repository that can combine data from multiple schools has unlimited possibilities as far as the educational research questions that can be asked and answered with a large IRB reviewed dataset (8). For example, the use of Big Data techniques, such as data mining, predictive analytics, or natural language processing, allow for ongoing educational research such as the discovery of new patterns, improved prediction of educational outcomes, and more generalizable conclusions (19, 30). Further, each institution can create a repository housing its valuable educational research data that can be seamlessly shared across institutions, broadening the scope of information and stimulating sophisticated research in a variety of settings. Any compatible datasets across schools can be considered a sub-network of a particular research evaluation thus creating multiple networks of data, whether in undergraduate, graduate, or continuing medical education settings. These evaluations can be accessed and shared by all researchers in medical education for further analysis and elucidation of best practices, including as yet unimagined Big Data approaches, which is fundamentally impossible without a fully implemented repository (19).

Our search found only three examples of institutions using the repository approach to IRB in medical education research. It is possible that other repositories utilizing this approach exist, but either haven't been published or our search terms did not capture them. The repositories featured in this paper are all based in the United States, and thus follow US IRB guidelines. Our exploration of repositories outside the United States suggests that the registry approach may not be currently available or applicable to educational research, as evidenced by what we learned about IRB processes in both Australia and Canada (25, 31).

In conclusion, the data repository approach to ethical review for medical education research has many benefits with efficient data management and analysis, and seamless collaboration within and across institutions. Having a repository that can combine data from multiple schools has unlimited possibilities as far as the educational research questions that can be asked and answered.
